# Food security and its association with socioeconomic status and dietary diversity in free living older people in Tehran, Iran

**DOI:** 10.1186/s12877-024-04705-y

**Published:** 2024-02-02

**Authors:** Fatemeh Pourebrahim, Nasrin Omidvar, Arezoo Rezazadeh, Hassan Eini-Zinab, Pedram Shirani, Delaram Ghodsi

**Affiliations:** 1 Department of Community Nutrition, Faculty of Nutrition Sciences and Food Technology, National Nutrition and Food Technology Research Institute, Shahid Beheshti University of Medical Sciences, No 46, Hafezi Street, Farahzadi Boulevard, Sharak Ghods, P.O. Box: 1981619573, Tehran, Iran; 2 Department of Nutrition Research, National Nutrition and Food Technology Research Institute, Faculty of Nutrition Sciences and Food Technology, Shahid Beheshti University of Medical Sciences, Tehran, Iran

**Keywords:** Food Security, Older People, USHFSSM, Dietary Diversity Score, Socioeconomic Factors, Iran

## Abstract

**Background:**

Food security is a function of food access and dietary diversity. Older age is a period when adequate and diverse dietary intake is a challenge. This study aimed to investigate the association between food security on the one hand and dietary diversity and socioeconomic factors on the other hand among the free-living older people in the city of Tehran.

**Methods:**

In this cross-sectional study, 583 older people, aged 60–80 years living in Tehran city, were selected through the systematic cluster sampling method. Food security was determined by the United States Household Food Security Survey Module (US-HFSSM (.Socioeconomic status (SES) and two 24-h recalls were obtained. Dietary Diversity Score (DDS) was calculated using the FAO 2010 guideline. Multinomial logistic regression was applied.

**Results:**

The average age of participants was 67.87 ± 5.86 years. Based on US-HFSSM, 56.9% of older people were food secure; while 25.7%, 14.2% and 3.2% suffered from food insecurity (FI) without hunger, with moderate hunger, and with severe hunger, respectively. There was no association between FI and DDS, even after controlling for confounders. FI with mild hunger was associated with household income (OR = 2.744, 95% CI = 1.100–6.846), while FI with severe hunger was associated with Fars ethnicity (OR = 0.146, 95% CI = 0.051–0.424).

**Conclusions:**

Overall, socio-economic status and demographic characteristics were the predictors of FI among older people. The findings can have implication in design and targeting of interventions directed at older people.

## Background

In recent decades, increasing life expectancy and decreasing fertility and birth rates have increased the proportion of older people in the general population and this trend will continue over the next decades [1]. The World Health Organization estimates that by 2050, older people population or those crossing the age of sixty will reach 2.1 billion (https://www.who.int/news-room/fact-sheets/detail/ageing-and-health). In Iran, as a country experiencing population transitions due to health and education developments, the population of older people, aged 60 and above, is growing significantly. The percentage of older people population in 2011 was 8.3%; however, it is expected to reach 10.3% in 2025 [2]. With the growing population of older people, the issue of health and well-being in the society is finding new and wider dimensions every day [3]. For all those who survive life events and go through youth and middle age, this time will come true. Therefore, ensuring health and well-being in this age group and improving their quality of life has become a priority from public health, as well as economic planning point of view.

Food security is one of the most important social, economic and political issues in different countries of the world, especially in middle and low income countries [4, 5]. Food Security, as defined by the Food and Agriculture Organization (FAO), is the situation that all people have access to adequate, healthy and nutritious food at all times in order to meet their nutritional needs and preferences for an active and healthy life [6, 7]. Food insecurity and hunger are of particular interest to researchers and policymakers because of the direct and indirect costs that they bring to individuals and therefore to society [8]. Among older people, in addition to old age, living alone, being disabled or physically incapable of doing things, especially buying and preparing food, and suffering from certain diseases, make them more vulnerable to food insecurity than younger individuals [7].

Food security affects food choices and food intake [8, 9], as well as diet diversity [10]. Diet diversity refers to the number of food items consumed over a period of time [11]. A diverse diet is associated with a higher intake of micronutrients [12]. Socioeconomic and demographic factors, including household size, house ownership, educational levels, access to health and insurance services, and total household income are the most important factors affecting food security [13, 14]. Studies have shown that there is a positive relationship between dietary diversity and access to energy and food at both individual and household levels [15]. It has been shown that increasing income in large cities is a factor in increasing individual diet diversity [16].

Several studies have examined the relationship between food security and food diversity and also between socioeconomic factors and food security among older people [17–19]. They found that low income and low educational level were the most important predictors of older people food insecurity (FI) in the world [18, 19]. A study examining the relationship between food security and dietary diversity in people over the age of 40 in a district in Tehran city found that Dietary Diversity Score (DDS), as one of the indicators of healthy eating was associated with food security status; and as the level of food security increased, DDS, especially fruits and vegetables DDS increased [17]. In another study in Shiraz (central Iran), an inverse relationship between food insecurity and consumption of meat, milk, fruits, and vegetables scores was reported [20]. Finally, a study on food insecurity and some associated risk factors in Tehranian older women showed that food insecurity was more frequent among women who were single, had swallowing problem and those with low to moderate socioeconomic status [21].

Nutrition and health status of older people are different from other age groups in the society, due to their higher vulnerability to diseases and adverse health conditions [14]. Given the prevalence of food insecurity and its risk factors in this age group and its impact on food intake and dietary diversity, it is important to identify major determinants of food security in older people and its association with diet quality. Few studies have examined food security status of the free-living older people in Iran and specifically in urban settings. According to the latest census, Tehran, the capital and the largest city of the country, has a population of 12,183,391 people, of whom 1,200,123 are over 60 years of age [4]. The present study aimed to investigate the relationship between food security and socioeconomic factors and dietary diversity among older people living in Tehran city.

## Methods

### Participants

This study was part of a large project entitled "Situation analysis of lifestyle of free-living older people residents of Tehran". In this cross-sectional study, 583 (304 Women, 279 men) free living older people residing in Tehran city were selected in 2017–2018 from those who were willing to participate in the study, were not residence in nursing homes or any institution, had Iranian nationality, aged between 60 and 80 years, with no severe illness or metabolic diseases such as cancer, end-stage kidney disease and severe metabolic and cognitive disorders, and were able to speak and communicate.

The number of subjects required was calculated with regard to food security status and dietary diversity of older people as the main variables, using Qomi et al. study in district 13 of Tehran [17]. Using this information and by adding Power of 90%, the calculated sample size was 446, and with counting 10% of the sample drop out, it was increased to 490; finally, to increase the accuracy of the study the sample size of 583 was considered.

The participants were selected by clustered systematic multi-stage sampling from public health centers (PHCs) [60% of the sample size]), community centers (called *Saraye-Mahalleh*) [20% of the sample] and mosques communities [20% of the sample] according to the demographic weight of the selected districts. Tehran districts were categorized into four zones, based on socio-economic factors [22]. Based on this ranking, districts 1, 3, 6 and 7 were ranked as more developed zone, districts 2, 4, 5, 8, 11, 12, 13, and 14 as medium-upward developed zone, districts 9, 15, 19, 20, 21 as medium-downward developed and districts 10, 16, 17, 18 and 22 as least developed zones [22].

### Measurements

#### Demographic and socioeconomic characteristics

For each older person, socio-economic characteristics were assessed through face-to-face interview, using a questionnaire [23]. The questionnaire consisted of demographic information, including age, gender, ethnicity (Fars, Azeri and others), marital status (single, married, widowed), relationship to the head of the household (head, spouse and other), number of children, as well as socio-economic characteristics: educational level (illiterate and elementary, middle and high school, High school diploma and higher), household size, employment status (unemployed, employed, housewife, retired and other), Household monthly income in US dollars (< 74, 74–148.15, 148.15–222.22, > 222.22), district of residence, home ownership status (owner, other), and welfare index (house area in m^2^, number of rooms, living facilities e.g., vehicle, television, freezer, refrigerator, gas, etc., and receiving food aids or financial support from organizations and charities).

#### Food security

Food security of the studied subjects was assessed using US-HFSSM questionnaire validated for Iranian older people by Rafiei et al. [24]. The US-HFSSM questionnaire includes 10 items that measure the levels of insecurity and occurrence and non-occurrence and the frequency of recurrence of the problem in the last 12 months [25]. The first question contained three response options, including often (occurrence per month), sometimes (occurrence in some months) and never/rarely (non/one or two months of the year). In two questions (number 4B and 8B), frequency of occurrence were asked in the form of almost every month, some months or up to two months in the past 12 months; the rest of the questions were yes/no questions. These questions were related to three different areas of access to food, including worrying and uncertainties about food stocks, inappropriate quality, low diversity and mismatch with food preferences and getting insufficient food [25].

#### Dietary intake

Dietary intake of the subjects was assessed using two non-consecutive 24-h recalls (one weekday and one weekend) by multiple-pass method (26) through interview by trained nutritionists using a questionnaire. The subjects were asked to estimate the amount of foods consumed, using home scales, geometric shapes, rulers, food models, and a food album [26]. If all the answers were not ready, they were interviewed by phone on the next day to complete the information [27].

#### Dietary diversity score

Using the data from the 24-h recalls, the FAO (2013) Dietary Diversity Score (DDS) was calculated [8]. Based on this approach, all foods were divided into 9 food groups: 1) cereals, 2) milk and dairy products, 3) vitamin A-rich fruits and vegetables, 4) green leafy vegetables, 5) other fruits and Vegetables, 6) Meat, fish and seafood, 7) Organ meats, 8) Eggs, and 9) Nuts, seeds and beans. DDS was calculated by consuming at least 15 g of one food item for each of the listed food groups during the two reported days. DDS was the total score of the food groups and ranged from 0 to 9 which was classified into two levels: low (≤ 3) and high (> 3) [28].

### Statistical analysis

Data were analyzed by SPSS software, version 21. After data cleaning, the normality test was used to determine if the distribution of variables is normal. The number of rooms and house dimension were divided by household size and their per capita value was calculated, separately. Also, each home living facility was scored 1 to 10, based on its financial weight. The total score was calculated as “Facility Score”. Finally, the sum of per capita number of rooms, per capita house size and facility score were defined as “welfare index”.

For calculating DDS, those with incomplete 24-h recall questionnaires and/or over or under-reporting of energy (below 500 and above 3500 kcal in women, below 800 and over 4000 kcal in men) 21 were excluded from the study (21 subjects).

Dietary intake, food security status, demographic characteristics and SES were analyzed by descriptive statistics. To test the association between independent and dependent variables, multinomial and binary logistic regression tests were used. Level of significance was considered as *P* < 0.05.

## Results

### General characteristics of the sample

Demographic characteristics of the study participants are presented in Table 1. Overall, 583 older people participated in this study with mean age of 67.87 ± 5.86 years of whom 304 (52.1%) were women. The average household size was 3.01 ± 1.41, and total number of children and number of children living with older people were, 3.83 ± 1.75 and 0.94 ± 1.05, respectively.

**Table 1:** Demographic characteristics of older people by sex, Tehran, Iran (*N* = 583)

		Male (*n* = 279) N (%)	Female (*n* = 304) N (%)	Total (*n* = 583) N (%)	*p*-value
Age (year)		69.54 ± 6.14^a)^	66.34 ± 5.14^a)^	67.87 ± 5.86^a)^	< 0.0001^*^
Total number of children		3.78 ± 1.78^a)^	3.89 ± 1.72^a)^	3.83 ± 1.75^a)^	0.562
Number of children living with older people		1.03 ± 1.12^a)^	0.85 ± 0.96^a)^	0.94 ± 1.05^a)^	0.044^*^
Living arrangement	Living alone	14 (5.0)	54 (17.8)	68 (11.7)	< 0.0001^*^
	With family	265 (95.0)	250 (82.2)	515 (88.3	
Marital status	Married	264 (94.6)	200 (65.8)	464 (79.6)	< 0.0001^*^
	Single	15 (5.4)	104 (34.2)	119 (20.4)	
Ethnicity	Fars	144 (51.8)	184 (60.7)	328 (56.5)	0.006^*^
	Azeri	76 (27.3)	50 (16.5)	126 (21.7)	
	Others	59 (20.9)	70 (22.8)	127 (21.8)	
Relationship with the head of the household	Head	276 (98.9)	117 (38.5)	393 (67.4)	< 0.0001^*^
	Spouse	3 (1.1)	187 (61.5)	190 (32.6)	

^a^Mean ± SD (Standard deviation)

^*^Significant

Socio-economic characteristics of the participants are shown in Table 2. About half were retired (44.9%) of whom most were men (74.6%). The percentage of women with monthly household income less than 74 US$ was almost twice (23.2% vs. 10.0%) older men; while the percentage of men with monthly household income of 148.15 to 222.22 US$ were about twice women. No sex differences was observed in the mean welfare index and household size.

**Table 2:** Socio-economic characteristics by sex of the participants, Tehran, Iran (*N* = 583)

		Male (*n* = 279) N (%)	Female (*n* = 304) N (%)	Total (*n* = 583) N (%)	*p*-value
Education level	Illiterate/elementary	138 (49.5)	166 (54.6)	304 (52.1)	0.228
	Middle/high/diploma	94 (33.7)	101 (33.2)	195 (33.4)	
	Bachelor or higher	47 (16.8)	37 (12.2)	84 (14.5)	
Employment status	Employed	35 (12.5)	4 (1.3)	39 (6.7)	< 0.0001*
	Retired	208 (74.6)	54 (17.8)	262 (44.9)	
	Housewives	5 (1.8)	240 (78.9)	245 (42.0)	
	Unemployed	21 (7.5)	3 (1.0)	24 (4.2)	
	Other	10 (3.6)	3 (1.0)	13 (2.2)	
House ownership status	Owner	237 (85.2)	253 (83.4)	490 (84.3)	0.642
	Tenant	42 (14.8)	51 (16.6)	93 (15.7)	
Area of residence	more-developed	77 (27.6)	94 (30.9)	171 (29.3)	0.182
	medium-upward developed	71 (25.5)	74 (24.3)	145 (24.9)	
	medium-downward developed	92 (36.9)	103 (30.3)	195 (33.4)	
	least-developed	28 (10.0)	44 (14.5)	72 (12.4)	
Household monthly income (US$)	< 74	29 (10.0)	71 (23.2)	100 (16.9)	< 0.0001*
	74–148.15	121 (43.8)	149 (49.2)	270 (46.7)	
	148.15–222.22	71 (25.5)	42 (13.8)	113 (19.5)	
	> 222.22	58 (20.7)	42 (13.8)	100 (16.9)	
Household size		3.15 ± 1.37^a^	2.88 ± 1.44^a^	3.01 ± 1.41^a^	0.619
Welfare index		63.23 ± 31.33^a^	64.42 ± 29.05^a^	63.86 ± 30.14^a^	0.479

^a^Mean ± SD (Standard deviation)

^*^Significant

### Food security and diet diversity status

Food security status and DDS of the participants are presented in Table 3. After exclusion of misreports, dietary diversity score was calculated for 511 participants. More than half of the participants were food secure (56.9%). Only 17.4% of participants reported food insecurity (FI) with hunger in the past 12 months. About three quarters of the participants had high dietary diversity score (74.6%). There was no significant differences between men and women in terms of FI and DDS.

**Table 3:** Food security status and dietary diversity score (DDS) by sex of older people participants, Tehran, Iran (*n* = 583)

Variable		Male (*n* = 279) N (%)	Female (*n* = 304) N (%)	Total (*n* = 583) N (%)	*p*-value
Food security status	Food secure	172 (61.9)	161 (52.8)	332 (56.9)	0.122
	Food insecure without hunger	67 (23.8)	83 (27.6)	150 (25.7)	
	Food insecure with moderate hunger	32 (11.0)	51 (16.3)	83 (14.2)	
	Food insecure with severe hunger	9 (3.3)	9 (3.3)	18 (3.2)	
DDS^a^	Low (≤ 3)	56 (23.4)	74 (27.2)	130 (25.4)	0.328
	High (> 3)	183 (76.6)	198 (72.8)	381 (74.6)	

^a^511 participants were analyzed

Based on Table 4, after adjusting for confounders in 2 models, the results of multinomial logistic regression indicated that being younger decreased the chance of food insecurity (FI) with moderate hunger (OR = 0.427, 95% CI = 0.206–0.886 for adjusted model with sex; OR = 0.317, 95% CI = 0.134–0.749 for adjusted model with other cofounders) compared to those aged 75 to 80 years. In addition, being Fars ethnic decreased the possibility of being food insecure (OR = 0.549, 95% CI = 0.324–931 for FI without hunger; OR = 0.146, 95% CI = 0.051–0.424 for FI with severe hunger) in comparison with other ethnicities. The number of children living with older people were associated with higher chance of FI without hunger and FI with moderate hunger in unadjusted model (OR = 1.171, 95%CI = 1.051–1.304) and (OR = 1.224, 95%CI = 1.072–1.396), respectively which diminished in the adjusted model. Sex, marital status, living arrangement and the number of children living with older people were not significantly associated with food insecurity.

**Table 4:** Unadjusted and adjusted association between demographic characteristics and food security status^d^ among older people living in Tehran, Iran (*N* = 583)

**FS** (*n* = 330)	**FI without hunger** (*n* = 148)			**FI with moderate hunger** (*n* = 81)			**FI with severe hunger** (*n* = 24)		
1.00 Ref	OR (95% CI)^a^ unadjusted	OR (95% CI) adjusted^b^	OR (95% CI) adjusted^c^	OR (95% CI)^a^ unadjusted	OR (95% CI) adjusted^b^	OR (95% CI) adjusted^c^	OR (95% CI)^a^ unadjusted	OR (95% CI) adjusted^b^	OR (95% CI) adjusted^c^
**Age** (years)									
60–65	0.572 (0.324–1.010)	0.618 (0.342–1.115)	0.676 (0.346–1.323)	0.497 (0.248–0.998)*	0.427 (0.206–0.886)*	0.317 (0.134–0.749)*	0.870 (0.216–3.506)	0.908 (0.216–3.819)	1.237 (0.252–6.079)
66–75	0.663 (0.387–1.135)	0.696 (0.403–1.203)	0.675 (0.374–1.219)	0.609 (0.318–1.164)	0.551 (0.284–1.070)	0.417 (0.199–0.877)*	1.153 (0.312–4.258)	1.185 (0.315–4.456)	1.477 (0.364–5.995)
76–80	1.00 Ref	1.00 Ref	1.00 Ref	1.00 Ref	1.00 Ref	1.00 Ref	1.00 Ref	1.00 Ref	1.00 Ref
**Sex**									
Female	0.753 (0.510–1.113)	0.897 (0.368–2.184)	0.653 (0.347–1.231)	1.249 (0.762–2.047)	1.467 (0.873–2.464)	1.438 (0.644–3.212)	0.877 (0.370–2.078)	0.821 (0.547–1.232)	1.367 (0.297–6.301)
Male	1.00 Ref	1.00 Ref	1.00 Ref	1.00 Ref	1.00 Ref	1.00 Ref	1.00 Ref	1.00 Ref	1.00 Ref
**Marital status**									
Single	1.178 (0.727–1.909)	1.353 (0.799–2.292)	1.274 (0.650–2.500)	1.542 (0.874–2.723)	1.365 (0.735–2.535)	1.607 (0.744–3.471)	0.963 (0.315–2.944)	0.998 (0.999–2.301)	1.053 (0.245–4.535)
Married	1.00 Ref	1.00 Ref	1.00 Ref	1.00 Ref	1.00 Ref	1.00 Ref	1.00 Ref	1.00 Ref	1.00 Ref
**Ethnicity**									
Fars	0.512 (0.311–0.841)*	0.517 (0.314–0.853)*	0.549 (0.324–0.931)*	0.636 (0.341–1.186)	0.649 (0.346–1.217)	0.648 (0.330–1.272)	0.129 (0.047–0.353)*	0.130 (0.047–0.358)*	0.146 (0.051–0.424)*
Azeri	1.215 (0.687–2.148)	1.200 (0.674–2.134)	0.824 (0.443–1.532)	1.186 (0.575–2.449)	1.265 (0.608–2.634)	0.802 (0.363–1.770)	0.235 (0.064–0.866)*	0.228 (0.061–0.850)*	0.164 (0.041–0.654)*
Other	1.00 Ref	1.00 Ref	1.00 Ref	1.00 Ref	1.00 Ref	1.00 Ref	1.00 Ref	1.00 Ref	1.00 Ref
**Living arrangement**									
With family	0.821 (0.454–1.482)	0.775 (0.420–1.431)	0.900 (0.462–1.751)	0.866 (0.411–1.825)	1.025 (0.474–2.220)	1.031 (0.451–2.357)	1.237 (0.278–5.508)	1.247 (0.271–5.744)	1.469 (0.308–7.017)
Living alone	1.00 Ref	1.00 Ref	1.00 Ref	1.00 Ref	1.00 Ref	1.00 Ref	1.00 Ref	1.00 Ref	1.00 Ref
**Relationship to the head of the household**									
Spouse	0.710 (0.464–1.085)	0.812 (0.458–1.429)	0.878 (0.448–1.721)	0.726 (0.426–1.238)	0.525 (0.273–1.009)	0.406 (0.188–0.876)*	0.675 (0.257–0.771)	0.619 (0.183–2.093)	0.516 (0.130–2.048)
Head	1.00 Ref	1.00 Ref	1.00 Ref	1.00 Ref	1.00 Ref	1.00 Ref	1.00 Ref	1.00 Ref	1.00 Ref
**Number of children living with older people**	1.083 (0.901–1.301)	1.101 (0.913–1.328)	1.092 (0.894–1.333)	1.054 (0.837–1.329)	1.115 (0.882–1.410)	1.100 (0.855–1.417)	1.004 (0.661–1.526)	1.011 (0.660–1.550)	0.977 (0.630–1.313)
**Number of children not living with older people**	1.171 (1.051–1.304)*	1.165 (1.042–1.302)*	1.049 (0.923–1.193)	1.224 (1.072–1.396)*	1.200 (1.047–1.375)*	1.054 (0.897–1.238)	1.126 (0.890–1.426)	1.120 (0.879–1.428)	0.966 (0.738–1.264)

Ref., reference category

^a^Calculated by multinomial logistic regression. 95% CI: Confidence Interval of the 95%.OR: odds radio

^b^Adjusted for age and sex

^c^Adjusted for age, sex, household income per month, education level, employment status, living arrangement, residential area

^d^The reference group for food security status is food secure

^*^Significant, all statistical significance was tested at *P* < 0.05 level

As shown in Table 5, earning less than 74 US$ per month increased the risk of FI without hunger (OR = 4.240, 95%CI = 1.997–9.001) and FI with moderate hunger (OR = 3.163, 95%CI = 1.296–7.716) in comparison with those with monthly income of higher than 222.22 US$, respectively. In addition, those who lived in most-developed districts were at lower risk of food insecurity compared to other districts (OR = 0.426, 95%CI = 0.216–839 for FI without hunger; OR = 0.397, 95%CI = 0.162–0.971 for FI with moderate hunger). Being retired was associated with a decreased risk of FI without hunger and FI with moderate hunger after adjusting for age and sex (OR = 0.567, 95%CI = 0.325–0.989) and (OR = 0.569, 95%CI = 0.337–0.960), respectively. Multinomial Logistic regression models suggested that after adjusting for age and sex, being illiterate increased the possibility of different levels of FI (OR = 3/107, 95%CI = 1/672–5/775), (OR = 2/736, 95%CI = 1/261–5/935) and (OR = 6/193, 95%CI = 1/368–28/039), respectively compared to those with high school diploma and higher degrees. Those with elementary to middle school education were significantly at higher risk of FI without hunger (OR = 1/821, 95%CI = 1/150–2/883) and sever FI (OR = 3/813, 95%CI = 1.073–13.546) in comparison with bachelor or higher education. House ownership status, household size and welfare index were not associated with FI.

**Table 5:** Unadjusted and adjusted association between socio-economic characteristics and food security^d^ status among older people living in Tehran, Iran (*N* = 583)

**FS** (*n* = 330)	**FI without hunger** (*n* = 148)			**FI with moderate hunger** (*n* = 81)			**FI with severe hunger** (*n* = 24)		
1.00 Ref	OR (95% CI)^a^ unadjusted	OR (95% CI) adjusted^b^	OR (95% CI) adjusted^c^	OR (95% CI)^a^ unadjusted	OR (95% CI) adjusted^b^	OR (95% CI) adjusted^c^	OR (95% CI)^a^ unadjusted	OR (95% CI) adjusted^b^	OR (95% CI) adjusted^c^
**Education level**									
Illiterate	3.224 (1.770–5.872)*	3.107 (1.672–5.775)*	1.662 (0.783–3.530)	3.155 (1.495–6.659)*	2.736 (1.261–5.935)*	1.156 (0.446–2.997)	5.877 (0.343–27.715)*	6.193 (1.368–28.039)*	3.632 (0.627–21.038)
Elementary to middle school	1.821 (1.150–2.883)*	1.805 (1.131–2.880)*	1.175 (0.676–2.042)	1.892 (1.055–3.395)*	1.795 (0.992–3.248)	0.915 (0.452–1.852)	3.813 (1.073–13.546)*	3.867 (1.076–13.903)*	2.905 (0.675–12.504)
Diploma or higher	1.00 Ref	1.00 Ref	1.00 Ref	1.00 Ref	1.00 Ref	1.00 Ref	1.00 Ref	1.00 Ref	1.00 Ref
**Employment status**									
Employed	0.936 (0.408–2.145)	0.640 (0.246–1.666)	0.778 (0.287–2.113)	1.123 (0.449–2.809)	1.423 (0.471–4.303)	1.633 (0.517–5.155)	1.476 (0.303–7.204)	1.384 (0.208–9.218)	2.390 (0.306–18.658)
Retired	0.887 (0.593–1.326)	0.567 (0.325–0.989)*	0.775 (0.406–1.479)	0.569 (0.337–0.960)*	0.539 (0.266–1.092)	0.611 (0.272–1.374)	0.969 (0.392–2.392)	0.862 (0.256–2.898)	1.981 (0.417–9.401)
Unemployed	1.00 Ref	1.00 Ref	1.00 Ref	1.00 Ref	1.00 Ref	1.00 Ref	1.00 Ref	1.00 Ref	1.00 Ref
**House ownership status**									
Tenant	1.347 (0.790–2.296)	1.374 (0.804–2.348)	1.344 (0.755–2.393)	0.924 (0.444–1.923)	0.949 (0.454–1.983)	0.975 (0.449–2.119)	1.021 (0.290–3.591)	1.017 (0.289–3.581)	0.942 (0.255–3.482)
Owner	1.00 Ref	1.00 Ref	1.00 Ref	1.00 Ref	1.00 Ref	1.00 Ref	1.00 Ref	1.00 Ref	1.00 Ref
**Household income per month** (US$)									
< 74	3.827 (1.839–7.965)*	4.240 (1.997–9.001)*	2.049 (0.883–4.751)	3.163 (1.296–7.716)*	2.744 (1.100–6.846)*	1.131 (0.397–3.224)	3.349 (0.588–19.059)	3.858 (0.658–22.622)	1.955 (0.289–13.222)
74–148.15	2.571 (1.358–4.870)*	2.637 (1.379–5.043)*	1.737 (0.871–3.467)	1.944 (0.887–4.264)	1.738 (0.784–3.855)	1.052 (0.441–2.512)	3.750 (0.835–16.845)	4.051 (0.889–18.466)	2.562 (0.525–12.497)
148.15–222.22	1.891 (0.908–3.937)	1.840 (0.881–3.842)	1.424 (0.663–3.060)	1.882 (0.780–4.544)	1.845 (0.762–4.467)	1.314 (0.519–3.328)	0.529 (0.047–5.973)	0.521 (0.046–5.888)	0.396 (0.034–4.606)
> 222.22	1.00 Ref	1.00 Ref	1.00 Ref	1.00 Ref	1.00 Ref	1.00 Ref	1.00 Ref	1.00 Ref	1.00 Ref
**Residential area**									
Most-developed	0.426 (0.216–839)*	0.406 (0.205–0.805)*	0.538 (0.263–1.100)	0.380 (0.164–0.881)*	0.366 (0.157–0.858)*	0.397 (0.162–0.971)*	0.456 (0.073–2.830)	0.459 (0.074–2.857)	0.723 (0.111–4.706)
Medium-upward developed	0.661 (0.337–1.297)	0.618 (0.313–1.222)	0.687 (0.339–1.391)	0.482 (0.204–1.137)	0.460 (0.192–1.098)	0.422 (0.168–1.055)	1.446 (0.278–7.278)	1.452 (0.287–7.325)	2.062 (0.394–10.788)
Medium-downward developed	1.525 (0.810–1.869)	1.501 (0.793–2.840)	1.493 (0.779–2.861)	1.525 (0.718–3.238)	1.640 (0.764–3.520)	1.641 (0.753–3.575)	2.346 (0.490–11.234)	2.371 (0.492–11.427)	2.247 (0.457–11.046)
Least-developed	1.00 Ref	1.00 Ref	1.00 Ref	1.00 Ref	1.00 Ref	1.00 Ref	1.00 Ref	1.00 Ref	1.00 Ref
**Household size**	1.002 (0.873–1.151)	1.011 (0.876–1.166)	1.025 (0.873–1.204)	1.027 (0.865–1.219)	1.067 (0.898–1.269)	1.088 (0.893–1.324)	0.949 (0.691–1.303)	0.953 (0.688–1.321)	0.912 (0.634–1.313)
**Welfare index**	0.999 (0.993–1.006)	0.999 (0.992–1.006)	1.000 (0.993–1.007)	0.999 (0.990–1.007)	0.999 (0.990–1.007)	0.997 (0.988–1.007)	0.993 (0.977–1.010)	0.993 (0.977–1.010)	0.995 (0.978–1.012)

Ref., reference category

^a^Calculated by multinomial logistic regression. 95% CI: Confidence Interval of the 95%.OR: odds radio

^b^Adjusted for age and sex

^c^Adjusted for age, sex, household income per month, education level, employment status, living arrangement, residential area

^d^The reference group for food security status is food secure

^*^Significant, all statistical significance was tested at *P* < 0.05 level

### Association between FS and DDS

Table 6, presents the results from unadjusted and adjusted binary regression analyses of the associations between dietary diversity score (DDS) and food security status. Older people who were food insecure with severe hunger had less odds to be in high DDS group; however, the association was non-significant.

**Table 6:** Unadjusted and adjusted association between dietary diversity score (DDS) with food security status^d^ among older people living in Tehran, Iran (*N* = 511)

	DDS	High (> 3) (*n* = 385)
**FS**		1.00 Ref
**FI without hunger**	OR (95% CI) unadjusted	0.771 (0.254–2.341)
	OR (95% CI) adjusted^b^	0.915 (0.291–2.881)
	OR (95% CI) adjusted^c^	1.384 (0.402–4.762)
**FI with moderate hunger**	OR (95% CI) unadjusted	1.073 (0.227–5.067)
	OR (95% CI) adjusted^b^	1.500 (0.304–7.406)
	OR (95% CI) adjusted^c^	2.802 (0.464–16.919)
**FI with severe hunger**	OR (95% CI)^a^ unadjusted	0.275(0.056–1.360)
	OR (95% CI) adjusted^b^	0.250 (0.050–1.266)
	OR (95% CI) adjusted^c^	0.263 (0.047–1.468)

Ref., reference category

^a^Calculated by binary logistic regression. 95% CI: Confidence Interval of the 95%.OR: odds radio

^b^Adjusted for age and sex

^c^Adjusted for age, sex, household income per month, education level, employment status, living arrangement, and residential area

^d^The reference group for food security status is food secure

## Discussion

The results of the present study showed a significant association between the level of household income and the severity of FI among the urban free-living older people, suggesting that income is one of the important associated factors with food security status in this age group. Other factors that were associated with the reduced risk of FI without hunger were being retired, being Fars (the dominant ethnic group in the country) and living in affluent districts of the city. Factors that were associated with reduced risk of FI with moderate hunger included age (being younger), living in most-developed areas, not being the head of the household and fewer number of children living with older people. The only factor that was associated with reduced risk of FI with severe hunger was ethnicity (being Fars or Azeri compared to other ethnics). There was no association between FI and DDS, even after controlling for confounders.

The findings are consistent with other studies that have suggested a relationship between income and food insecurity [29–31]. A direct relationship between income changes and changes in food sufficiency has been shown in a study in Michigan [29], where changes in income and employment status were related to changes in severity of food insecurity, as measured by HFSSM. Older people who received lower income faced 4 times higher odds of being FI without hunger and 2 times higher odds of being FI with mild hunger compared with those who had sufficient income. These findings suggest that income has a crucial role in sustaining food security for older people. Effects of adequate income on food security have been noted in the existing literatures [18, 19, 21, 32, 33]. However, this study found that there was not significant relationship between lower income and FI with severe hunger. The voluminous number of positive responds on the HFSSM module has been used as outcome variable in other studies [34–37]. This study also showed that a change in individual food insecurity scale score is an important indicator of economic changes within households.

Another unique finding was that illiterate older people and those with less than secondary education faced 6 times and more than 3 times higher odds of being FI with severe hunger compared to those with Bachelor or higher education, respectively. In fact, the highest prevalence as well as the greatest severity of food insecurity was observed among illiterate subjects. This could reflect an important limitation for illiterate older people to have higher level jobs and therefore have less income. Besides, getting any or additional job and increasing work hours may not be possible for many older people, particularly those with low educational levels.

The findings suggest that being retired reduced the chance of food insecurity among older people compared with those who were unemployed or their previous jobs did not provide retirement. The relationship between a gain in employment and reduction in severity of food insecurity have been observed in other studies, as well [32–34]. Retired seniors are probably better off financially compared to people who still have to work at this age.

Whereas previous studies showed that older people, with more children living with them were more vulnerable to food insecurity [18, 38], in the current study, those who had children not living with them were at higher risk of food insecurity. Although household size and marital status was not related with food security in the present study, a recent study of food security among households explained that households with single women, larger household size, and low incomes were more likely to be food insecure [39]. Older people are poorer physically and functionally than younger ones, affecting their ability to cook, consume and absorb foods. In addition, factors such as living alone or with fewer children, as a sign of being alone or lack of support can lead to increased chances of food insecurity in this age group [18, 40, 41].

Respondents in the most-developed residential districts were significantly less likely to experience food insecurity even in the mild form. Although a cross-sectional study in Australia did not find significant association between food insecurity and residential area [18], most studies approve the fact that living in wealthy areas was negatively associated with FI risk [19, 42, 43].

The present results showed that the risk of food insecurity was lower if older people were Fars and Azeri. A cross-sectional study which was done in Arak, central Iran, also supports this observation [32]. The relationship between being Azeri and reduction the risk of food insecurity has been observed in other studies in Iran [42]. A study has shown that Azeri people have a good social economic status [42, 44]. Higher food security of Fars people was due to being the dominant ethnic group, as well as being Azeri who are likely to be immigrants with longer residence time, better job prospects and higher life expectancy compared to other ethnicities.

The present study showed that marital status and gender were not related to older people’s food security status, even after adjusting for cofounders. Other studies also found no association between marital status and gender with food security status in older people [18, 32].

Although the relationship between dietary diversity and food security status was not significant in the present study, several studies have found this inverse association to be significant [43, 45]. A study in Iran has shown that the mean DDS of participants in the high food security group was significantly higher than the food insecure group [17]. A cross-sectional study in Taiwan showed that dietary diversity score was negatively associated with older people’s food security status, especially with regard to meat group DDS [46]. The lack of a significant relationship between food security status and DDS in this study, may be due to the fact that FAO (2013) instructions for calculating the DDS is easy without considering the servings of food groups consumption.

The relatively large sample of the present study provided an opportunity for investigating the association between socio-economic and demographic characteristics and severity of food insecurity. Also, it provided a useful snapshot of free living older people FI status and its approximate determinants. The very high response rate (583 participants) is also a notable strength of our study. However, due to the cross-sectional design of the study, we were unable to assess any bias that were from non-responders. The cross-sectional design also prevents an analysis of temporal association and causality. Since nutritional outcomes could inversely affect food security determinants, reverse causality is possible. Prospective monitoring of food security and its determinants is required to clarify the direction of causation. Also, the measures used do not quantify all hypothetical determinants; this limits the study in the comprehensiveness of the analysis and possibility of missing cofounders. Educational levels, employment status and other factors may be a product of financial and nonfinancial limitations directly related to food security and nutrition, and therefore the estimates of this study cannot be explained as cause and effect. US-HFSSM is limited its representation of ‘individual food security’ because it screen the respondents’ perception of whether they had enough food; HFSSM might only measure calorie, but not micronutrient.

## Conclusion

In conclusion, this study shows that income, employment status and educational levels of older people were the strongest predictors of food insecurity and its severity, highlighting the sensitivity of food-insecurity to socio-economic characteristics. These findings suggest that more household monthly income and better employment status of older people is associated with food security. The results support the need for development of public policy aimed at improving the basic resources of food-insecure older people to improve the quality of their life. Also, it calls for an instant need for development of a policy aimed at identifying and supporting food-insecure older people in Tehran and other metropolitans in the country, as currently there is no public policy in place to tackle this problem. Development of assistance programs for low-income and less educated older people is recommended. This study provides support for improving availability of secure employment opportunities and facilitating the retirement process for older people. No significant relationship between food security status and DDS was found.

## Data Availability

The datasets generated and analyzed during the current study are not publicly available due to the fact that the used data is part of an ongoing cohort and it is not possible to share the data to the public at this time but are available from the corresponding author on reasonable request.

## Abbreviations

US-HFSSM: United States Household Food Security Survey Module
SES: Socioeconomic Status
FI: Food Insecurity
FAO: Food and Agriculture Organization
PHCs: Public Health Centers
BMI: Body Mass Index
MAR: Mean Adequacy Ratio
NAR: Nutrient Adequacy Ratio
DDS: Dietary Diversity Scores
